# Palliative care practice and moral distress during COVID-19 pandemic (PEOpLE-C19 study): a national, cross-sectional study in intensive care units in the Czech Republic

**DOI:** 10.1186/s13054-022-04066-1

**Published:** 2022-07-19

**Authors:** Tereza Prokopová, Jan Hudec, Kamil Vrbica, Jan Stašek, Andrea Pokorná, Petr Štourač, Kateřina Rusinová, Paulína Kerpnerová, Radka Štěpánová, Adam Svobodník, Jan Maláska, RIPE-ICU study group, Jan Maláska, Kateřina Rusinová, David Černý, Jozef Klučka, Andrea Pokorná, Miroslav Světlák, František Duška, Milan Kratochvíl, Alena Slezáčková, Milan Kratochvíl, Petr Štourač, Tomáš Gabrhelík, Josef Kuře, Daniel Suk, Tomáš Doležal, Tereza Prokopová, Jana Čerňanová, Kamil Vrbica, Klára Fabiánková, Eva Straževská, Jan Hudec

**Affiliations:** 1 Department of Anaesthesiology and Intensive Care Medicine, Faculty of Medicine, Masaryk University and University Hospital Brno, Jihlavská 20, 625 00 Brno, Czech Republic; 2 Department of Simulation Medicine, Faculty of Medicine, Masaryk University, Kamenice 3, 625 00 Brno, Czech Republic; 3 Czech National Centre for Evidence-Based Healthcare and Knowledge Translation (Cochrane Czech Republic, Czech EBHC: JBI Centre of Excellence, Masaryk University GRADE Centre), Faculty of Medicine, Masaryk University, Kamenice 3, 625 00 Brno, Czech Republic; 4 Faculty of Medicine, Institute of Biostatistics and Analyses, Masaryk University, Kamenice 3, 625 00 Brno, Czech Republic; 5 Department of Health Sciences, Faculty of Medicine, Masaryk University, Kamenice 3, 625 00 Brno, Czech Republic; 6 Department of Paediatric Anaesthesiology and Intensive Care Medicine, Faculty of Medicine, Masaryk University and University Hospital Brno, Černopolní 9, 613 00 Brno, Czech Republic; 7 Department of Palliative Medicine, First Faculty of Medicine, Charles University and General University Hospital in Prague, Karlovo náměstí 32, 128 08 Prague, Czech Republic; 8 ANOVA CRO S.R.O., Prague, Czech Republic; 9 Department of Pharmacology, Medical Faculty of Masaryk University, Kamenice 3, 625 00 Brno, Czech Republic

**Keywords:** Palliative care, COVID-19, Moral distress, Pandemic, Inappropriate care, Ethical climate, Survey

## Abstract

**Background:**

Providing palliative care at the end of life (EOL) in intensive care units (ICUs) seems to be modified during the COVID-19 pandemic with potential burden of moral distress to health care providers (HCPs). We seek to assess the practice of EOL care during the COVID-19 pandemic in ICUs in the Czech Republic focusing on the level of moral distress and its possible modifiable factors.

**Methods:**

Between 16 June 2021 and 16 September 2021, a national, cross-sectional study in intensive care units (ICUs) in Czech Republic was performed. All physicians and nurses working in ICUs during the COVID-19 pandemic were included in the study. For questionnaire development ACADEMY and CHERRIES guide and checklist were used. A multivariate logistic regression model was used to analyse possible modifiable factors of moral distress.

**Results:**

In total, 313 HCPs (14.5% out of all HCPs who opened the questionnaire) fully completed the survey. Results showed that 51.8% (*n* = 162) of respondents were exposed to moral distress during the COVID-19 pandemic. 63.1% (*n* = 113) of nurses and 71.6% of (*n* = 96) physicians had experience with the perception of inappropriate care. If inappropriate care was perceived, a higher chance for the occurrence of moral distress for HCPs (OR, 1.854; CI, 1.057–3.252; *p* = 0.0312) was found. When patients died with dignity, the chance for moral distress was lower (OR, 0.235; CI, 0.128–0.430; *p* < 0.001). The three most often reported differences in palliative care practice during pandemic were health system congestion, personnel factors, and characteristics of COVID-19 infection.

**Conclusions:**

HCPs working at ICUs experienced significant moral distress during the COVID-19 pandemic in the Czech Republic. The major sources were perceiving inappropriate care and dying of patients without dignity. Improvement of the decision-making process and communication at the end of life could lead to a better ethical and safety climate.

*Trial registration*: NCT04910243.

**Graphical abstract:**

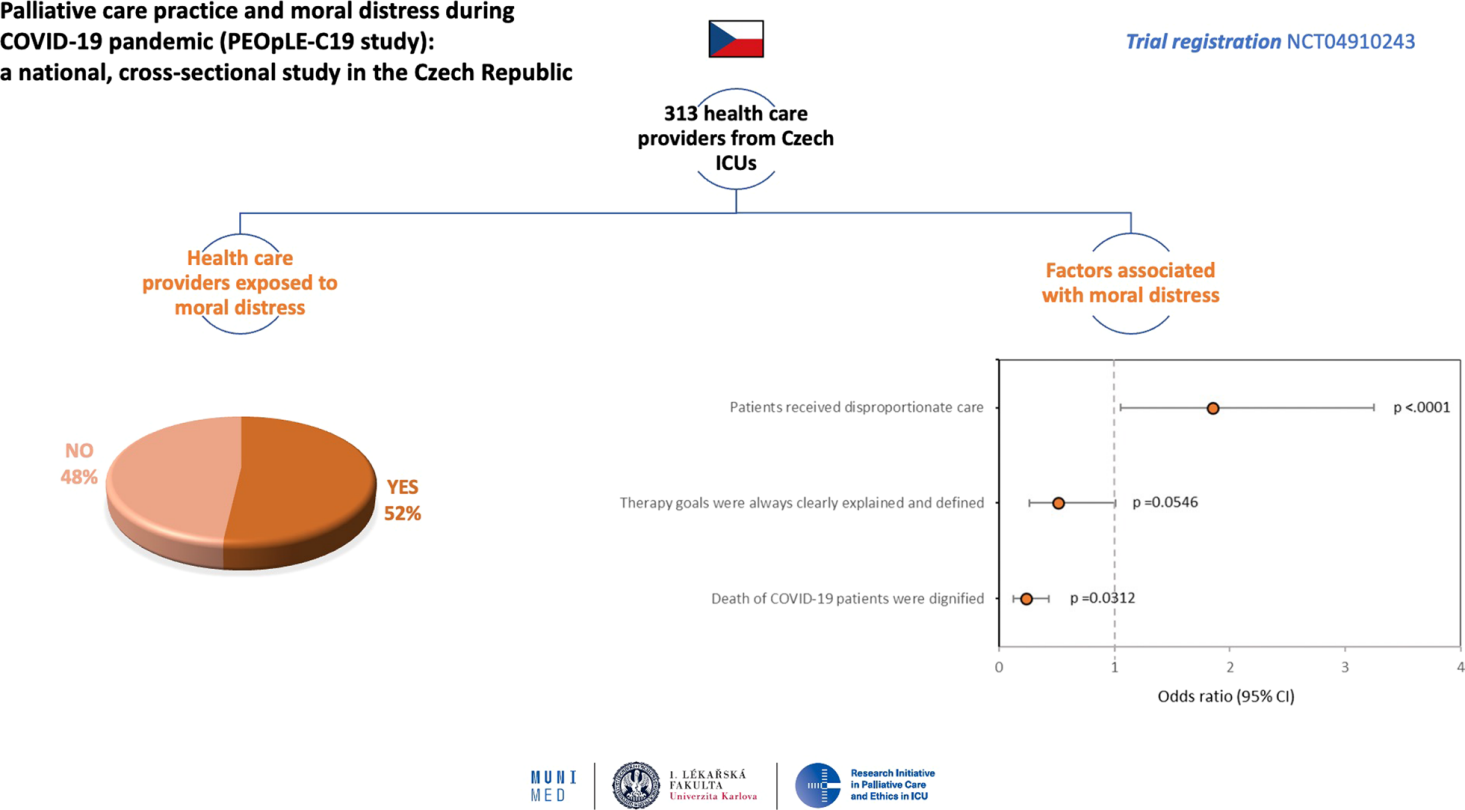

**Supplementary Information:**

The online version contains supplementary material available at 10.1186/s13054-022-04066-1.

## Background

Palliative care is active, complex, multidisciplinary, patient, and family-centred care addressing patients with serious and incurable diseases. Its aim is to improve the quality of life by relieving physical, psychological, social, and spiritual aspects of patients' suffering [1–3]. Palliative care, together with intensive care, is inseparable and overlapping parts of modern holistic care provided in the intensive care units (ICUs) [3]. In the Czech Republic, the palliative and end-of-life (EOL) care in ICUs is provided predominantly by ICU staff. The need for high-quality palliative care greatly increased in the recent months and years during the coronavirus disease 2019 (COVID-19) pandemic. This increased need was caused primarily by health care system overload with dozens of patients requiring multiple organ support with high mortality, especially among elderly patients with comorbidities [4–7]. Moreover, provided care in ICUs was frequently complicated by limited scarce resources, especially ICU staff and beds [8–11].

Principles of palliative care and EOL care are based on a consensus of many professional societies and supported by local recommendations and guidelines [12–14]. This standard for palliative care was severely challenged during the pandemic, through public policy restrictive measures separating families from patients by restricting visiting hours due to highly contagious disease with considerable mortality [15–18]. Despite the possibility of palliative care consultation by a dedicated palliative team, the COVID-19 pandemic led to a change in health care practice in many situations [13]. Overloading of health systems necessitated the involvement of health care providers (HCPs) in newly set-up ICUs without adequate experience or education in intensive care [9]. Hence, HCPs were under pressure and forced to prioritise due to lack of staff, equipment, or drugs, sometimes with limited policies by hospital officials or local professional societies or even without any of them [19–21]. These factors, including practice heterogeneity, probably triggered or worsened distress for most HCPs and potentially worsened the working environment with possible consequences for the quality of provided health care [19, 20, 22]. We hypothesised that the COVID-19 pandemic led to the differences in providing palliative care at the EOL and making end-of-life decisions (EOLDs) that could lead to distress within the HCPs team. The main aim of this observational, cross-sectional survey was to assess the practice of palliative care at the EOL in ICUs during the COVID-19 pandemic in the Czech Republic, the level of moral distress, and its possible modifiable factors.

## Methods

The Ethics Committee of the Faculty of Medicine of Masaryk University Brno approved this study on 16 June 2021 (No.61/2021). The PEOpLE-C19 survey was also registered on the ClinicalTrials.gov registry (NCT04910243). The questionnaire was developed by a team of experts from the Faculty of Medicine, Masaryk University Brno and the Faculty of Medicine, Charles University Prague that subsequently formed the RIPE-ICU study group (Research Initiative in Palliative Care and Ethics in the Intensive Care Unit). The survey was endorsed by The Ministry of Health of the Czech Republic and many professional societies (e.g. The Czech Society for Palliative Medicine, The Czech Society of Anaesthesiology and Intensive Care Medicine, The Czech Society of Intensive Care Medicine, The Czech Association of Nurses, The Association of University-educated Nurses, The Association of University Educators of Paramedical Health Professionals, The Czech Society of Surgeons, The Czech Pulmonological and Phthisiological Society). The survey link (https://is.muni.cz/pruzkum/22382) was circulated by official email of each society. Also, it was disseminated by social media (e.g. Facebook) to all members of endorsing professional societies. HCPs were invited to anonymously complete the survey open for three months (16 June to 16 September 2021). The inclusion criteria for participation were working in ICU during the COVID-19 pandemic in the Czech Republic either as a physician or a nurse. No other HCPs could participate. The survey was accessible only once from a unique IP address to prevent multiple participation. We obtained a significant amount of diverse data from this survey, and the complete results and source data are in Additional file 2: Appendix 2 and Additional file 3: Appendix 3, respectively. Only data about moral distress and characteristics of the COVID-19 pandemic practices are analysed and discussed in this article. The raw data and the analysis regarding practices of palliative and EOL care outside the COVID-19 pandemic in the Czech Republic could be found in Additional file 2: Appendix 2 and Additional file 3: 3, and the focused analysis will be published separately in a Czech peer-reviewed journal.

Inappropriate care was defined according to the DISPROPRICUS study as care that is no longer consistent with the expected survival or quality of life (either ‘too much’ or ‘not enough’ care), or that is provided against the patient’s or relatives’ wishes [30].

### Questionnaire development

The questionnaire was divided into four domains and consisted of 40 questions. The questionnaire development and complete list of questions are available in Additional file 1: Appendix 1. For the development of the questionnaire, the modified Delphi technique plus ACADEMY and CHERRIES guide and checklist described elsewhere was used [23–25]. Briefly, in the first step, the questionnaire items were generated through literature review and in-depth interviews with opinion leaders in palliative care and intensive care medicine and potential respondents. The second step was item reduction by limiting only relevant questions without eliminating potential domains. Eventually, grouping into domains was performed after no new items emerged, and the consensus was achieved in focus-group sessions. Validation was performed based on a specification table with research questions on the vertical axis and the domains on the horizontal axis. Items were reduced to the final questionnaire by an iterative process. Pretesting of this survey was performed by a group of invited experts from Masaryk University in Brno and Charles University in Prague. Each investigator read and approved the final version. The complete list of questions is presented in Additional file 1: Appendix 1. For publishing this survey, the CROSS checklist was used [26].

### Statistical analysis

Absolute and relative counts describe data from the survey for categorical parameters and median and interquartile range (IQR) for continuous parameters. Binomial logistic regression was performed to identify potential predictors of moral distress and to estimate odds ratios and 95% Wald CIs. Answers to the question ‘Were you exposed to moral distress during the COVID-19 pandemic?’ were grouped as follows: 'Strongly agree' or 'Somewhat 'agree' was grouped to ‘I was exposed to moral distress’; ‘Somewhat disagree’ or ‘Strongly disagree’ was grouped to ‘I was not exposed to moral distress’. Answers ‘Do not know’ occurred in a minority of cases and were not considered for this analysis. A univariate model was employed for each potential predictor of moral distress. Potential predictors significant at alpha level 0.1 were analysed via a multivariate logistic regression model. Parameters significant at alpha level 0.05 (*p* < 0.05) could be considered independent predictors of moral distress. *P* values should be considered exploratory, given the character of the analysis as well as the unbalanced groups compared. All statistical analyses were conducted using SAS 9.4 (SAS Institute, Cary NC).

## Results

Overall, 313 (14.5% out of all HCPs who opened the questionnaire) HCPs from the Czech Republic caring for COVID-19 patients fully completed the survey. The information about respondents' characteristics, e.g. their profession, position, and qualification, is presented in Table 1.

**Table 1:** Respondent’s characteristics and COVID-19 experience

	Results (*N* = 313)
Age, yr, median (IQR)	42 (33–48)
Sex, F, *n *(%)	207 (66.1)
*Profession of HCPs, n (%)*	
Nurse	179 (57.2)
General nurse	143 (79.9)
Paramedic	11 (6.1)
Children’s nurse	6 (3.4)
Practical nurse	5 (2.8)
Student	3 (1.7)
Midwife	3 (1.7)
Physician	134 (42.8)
*Position*	
Consultant	52 (38.8)
Senior Consultant	32 (23.9)
Head of the ICU	28 (20.9)
Resident	14 (10.4)
Head of the clinic	3 (2.2)
Qualification*	
Anaesthesia and intensive care medicine	92 (68.7)
Intensive care medicine	27 (20.1)
Internal medicine	18 (13.4)
Palliative care	14 (10.4)
Surgery	12 (9.0)
Infectious disease	8 (6.0)
Other	7 (5.2)
ICU experience, yr, median (IQR)	12 (3–20)
*COVID-19 ICU, n (%)*	
Established ICU	191 (61.0)
New COVID-19 ICU	100 (31.9)
*Type of ICU before COVID-19, n (%)*	
ICU in anaesthesia and intensive care medicine dpt	180 (57.5)
Patients/HCPs, *ratio*	3.15
Patients/Nurses, *ratio*	2.46
Patients/Physicians, *ratio*	4.08
*Type of hospital, n (%)*	
University (tertiary) hospital	145 (46.3)
Secondary hospital	104 (33.2)
Primary hospital	48 (15.3)
Comfort care experience before COVID-19, Yes, *n *(%)	264 (84.3)
Local protocol available, Yes, *n *(%)	97 (31.0)
Palliative consult available, Yes, *n *(%)	128 (40.9)
Advanced directives experience, Yes, *n *(%)	123 (39.3)
Advanced directives respected, Yes	97 (78.9)

*COVID-19* Coronavirus disease, *IQR* interquartile range, *ICU* intensive care unit, *HCPs* health care providers

*More than one answer possible

### Palliative care experience

**Fig. 1:**
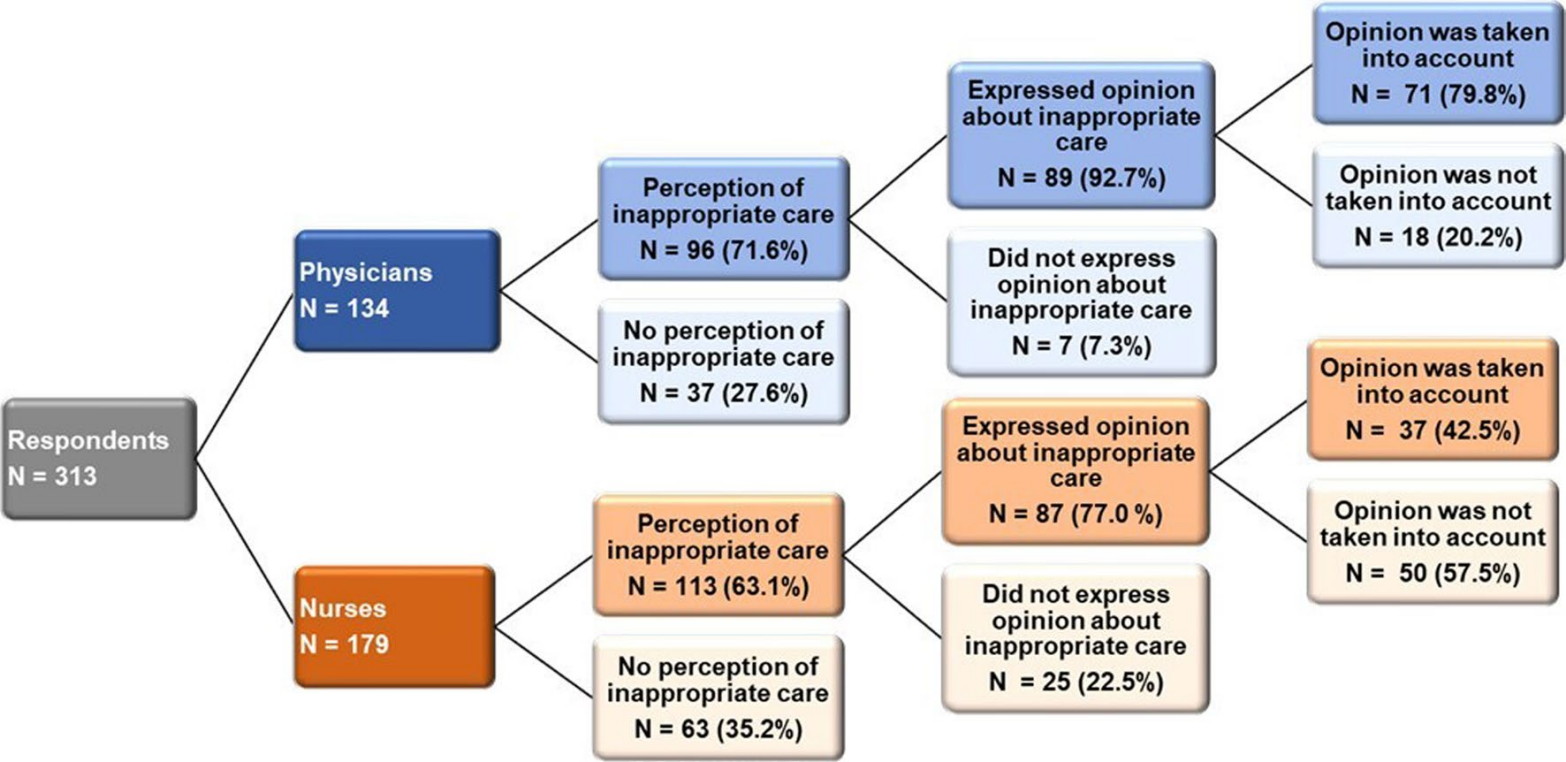
Expression and respect of HCPs opinion regarding perceiving disproportionate organ support

One hundred and seventy-six (56.2%) of respondents answered that palliative care was more demanding than full treatment (answer strongly agree and somewhat agree). Care of patients perceived as inappropriate was reported by 63.1% (*n* = 113) of nurses and 71.6% of (*n* = 96) physicians—organ support was reported as too long in 48.2%, and organ support was perceived as too extensive in 36.4%. 77.0% (*n* = 87) of nurses specifically expressed their opinion in the situation when organ support was perceived as inappropriate. In 57.5% (*n* = 50), this opinion was not taken into account. Physicians’ opinion about inappropriate care was respected in 79.8% (*n* = 71)—Fig. 1. The difference between nurses and physicians in expressing their opinion regarding inappropriate care and taking it into account was statistically significant (*p* < 0.001). The details of EOLD practice during the COVID-19 pandemic are described in Table 2. Patients’ dying was reported as not dignified (strongly and somewhat disagree) in 35.2% (*n* = 110). The factors for undignified death and reported factors of possible differences in palliative care before and during the COVID-19 pandemic are described in Table 3.

**Table 2:** Description of palliative care and EOLD during COVID-19 pandemic

	Results (*N* = 313)
Personnel involved in EOLD process*, *n* (%)	
Attending physician	293 (93.6)
Head of the ICU	253 (80.8)
Patient’s relatives	170 (54.3)
Attending nurse	157 (50.2)
Consultant physician	90 (28.8)
Matron/Head nurse	77 (24.6)
Palliative care physician	22 (7.0)
Form of EOLD communication with patient’s family*, *n* (%)	
Family meeting	269 (85.9)
Telephone call	203 (64.9)
Meeting with patient’s family was not performed	12 (3.8)
Videocall	9 (2.9)
Involvement of the patient's family regarding EOLD, *n* (%)	
Family/relatives were just informed about the decision; Patient was in comfort care if family/relatives did not disagree with the decision	141 (45.0)
Family/relatives were included in the process, i.e. shared decisions	81 (25.9)
Cannot answer as I was not involved in the decision process	39 (12.5)
Family/relatives were just informed about the decision; Patient was in comfort care despite family’s disagreement	33 (10.5)
Family/relatives were not informed about the decision	10 (3.2)
Family/relatives were fully responsible for the decision	7 (2.2)
Inappropriate care of patient*, *n* (%)	
Yes, organ support was too long	151 (48.2)
Yes, organ support was too extensive	114 (36.4)
No	100 (31.9)
Yes, organ support was not extensive enough	40 (12.8)
Yes, organ support was too short	36 (11.5)
Physician	
Express opinion about inappropriate care, Yes, *n* (%)^a^	89 (92.7)
Opinion respected, Yes^b^	71 (79.8)
Nurse	
Express opinion about inappropriate care, Yes, *n* (%)^a^	87 (77.0)
Opinion respected, Yes^b^	37 (42.5)
Resource scarcity situation experience, *n* (%)	
No	86 (27.5)
Yes, occasionally	36 (11.5)
Yes, repeatedly	10 (3.2)
* Did not answer*	181 (57.8)
Practice of comfort care was different during/before COVID-19 pandemic	
Strongly agree	49 (15.7)
Somewhat agree	104 (33.2)
Do not know	34 (10.9)
Somewhat disagree	90 (28.8)
Strongly disagree	32 (10.2)

*COVID-19* Coronavirus disease, *IQR* interquartile range, *EOLD* end-of-life decision

^a^Percentage based on number of physicians/nurses who considered care of patient as inappropriate

^b^*P* < 0.001 between nurses and physicians regarding expressing their opinion regarding inappropriate care and respecting opinion

*More than one answer possible

**Table 3:** Possible factors of different practices of palliative care during COVID-19 pandemic

	Results (*N* = 313)
Factors of different practice*, n (%)^*a*^	
Health system congestion	107 (69.9)
Personal factors	101 (66.0)
Primary nature of COVID-19 disease	90 (58.8)
Organisational and process factors	75 (49.0)
Technical equipment	69 (45.1)
Different ethical principles	44 (28.8)
Communication within the team	27 (17.6)
Process of EOLD discussions	22 (14.4)
Emotions	19 (12.4)
Communication with the management	18 (11.8)
*Therapy goals were always clearly explained and defined, n (%)*	
Strongly agree	60 (19.2)
Somewhat agree	168 (53.7)
Do not know	35 (11.2)
Somewhat disagree	35 (11.2)
Strongly disagree	7 (2.2)
*Most COVID-19 patients were dying with dignity, n (%)*	
Strongly agree	69 (22.0)
Somewhat agree	124 (39.6)
Do not know	35 (11.2)
Somewhat disagree	61 (19.5)
Strongly disagree	14 (4.5)
Factors which contributed to absence of dignity*, n (%)^*b*^	
System problems	52 (22.2)
Inconsistent opinions of physicians on comfort care	40 (17.1)
Principles of comfort care were not fully understood	29 (12.4)
Insufficient control of patient’s symptoms	21 (9.0)
Insufficient communication within the team	18 (7.7)
Inconsistent opinions of nurses on comfort care	18 (7.7)
Resource scarcity situation	7 (3.0)
*Doubts about EOLD process experience, n (%)*	
No, process was respecting medical and ethical principles	214 (68.4)
Yes, I did not consider process adequate	52 (16.6)
Yes, professional medical reasons	25 (8.0)
Yes, moral reasons	5 (1.6)
*Resource scarcity situation used as supporting argument in EOLD, n (%)*	
No	154 (49.2)
Yes, but I understood importance of the argument	108 (34.5)
Yes, but I was not comfortable with the argument	36 (11.5)

*COVID-19* Coronavirus disease, *EOLD* end-of-life decision

^a^Percentage based on number of HCPs who answered ‘Strongly agree’ or ‘Somewhat agree’ on the question ‘Principles and practice of comfort care differ during/before Covid-19 pandemic’

^b^Percentage based on number of HCPs who answered any option except for ‘Strongly agree’ on the question ‘Most COVID-19 patients were dying with dignity’

*More than one answer possible

### Moral distress

**Fig. 2:**
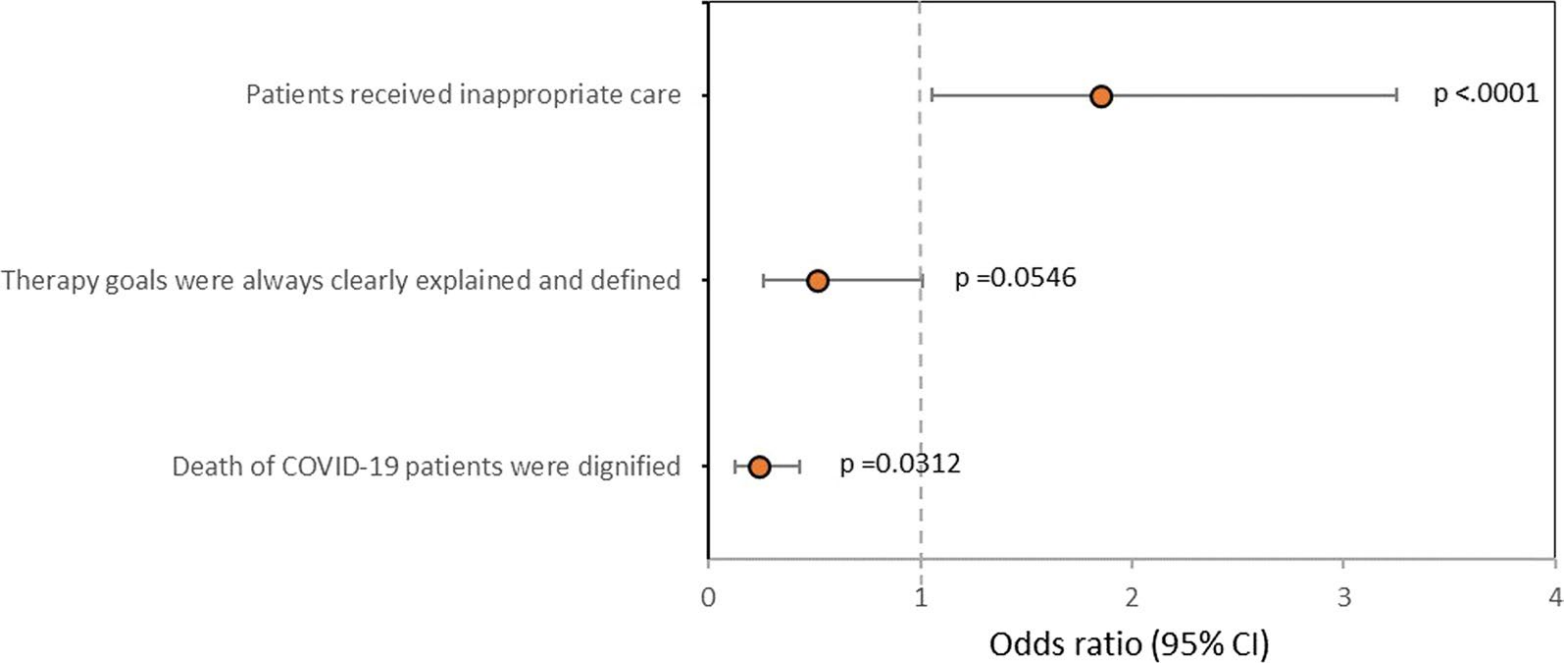
Factors associated with moral distress – multivariate analysis

**Fig. 3:**
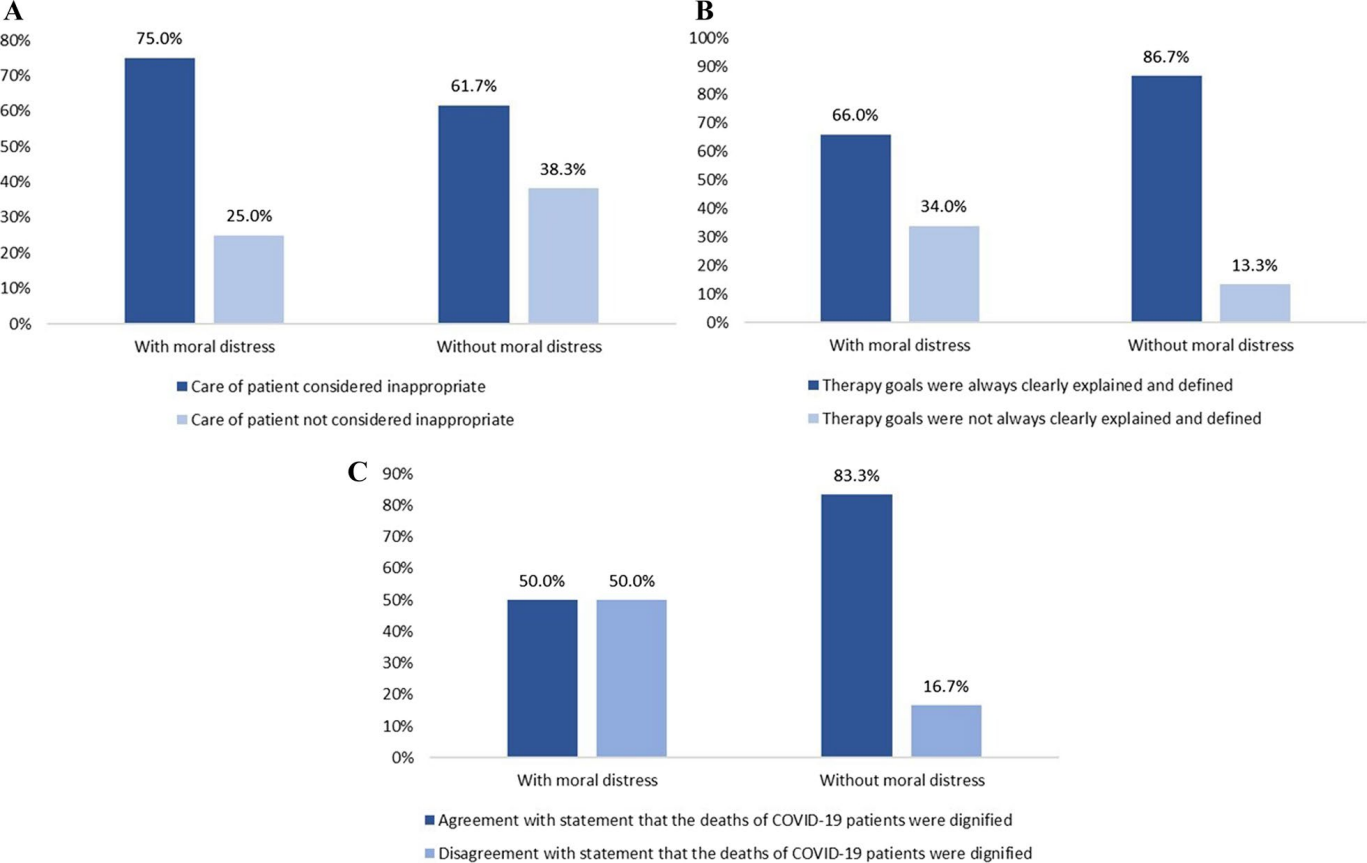
Bar charts depicting proportion of reported moral distress related to the predictors for moral distress

As shown in Table 4, reported major sources of moral distress were spending less time with patients (53.7%), inconsistency of opinions regarding transition, practice of palliative care by physicians (42.5%) and nurses (23.0%), followed by insufficient communication with patient’s family (23.6%) and within the ICU team (22.0%). In 51.8% (*n* = 162) of all respondents, moral distress was reported. Moral distress was comparable to the situation before the COVID-19 pandemic at 17.9% (*n* = 29) (somewhat agree and strongly agree). Nurses with ICU experience reported moral distress in 52.6% (*n* = 71) and nurses without ICU experience in 54.1% (*n* = 13). Potential predictors of moral distress analysed by univariate logistic regression are shown in Table 5 and the results of the logistic regression model in Table 6. In case the perception of inappropriate care was stated, there was a 1.8 × higher chance for the occurrence of moral distress for HCPs (OR, 1.854; CI, 1.057–3.252; *p* = 0.0312). Respecting HCPs’ opinion regarding inappropriate care tended to lower the chance for HCPs’ moral distress by about 50% (OR, 0.511; CI, 0.261–1.001; *p* = 0.0504). Patients’ dignified dying lessens the chance for experiencing moral distress by HCPs by about 76% (OR, 0.235; CI, 0.128–0.430; *p* < 0.001). Clear explanation of the therapy goals seems to be a potential predictor for reducing the chance for occurrence of HCP’s moral distress in univariate analysis (OR, 0.297; CI, 0.160–0.551; *p* < 0.001), but not proved as an independent predictor in the multivariate model. (*p* = 0.0546)—Fig. 2. Proportion of reported moral distress related to the potential predictors for moral distress is depicted in Fig. 3 (A-C).

**Table 4:** Distress and moral distress during COVID-19 pandemic

	Results (*N* = 313)
Major sources of distress*, *n* (%)	
Spending less time with patients	168 (53.7)
Inconsistent opinions of physicians regarding comfort care	133 (42.5)
Insufficient communication with patient’s family	74 (23.6)
Inconsistent opinions of nurses on comfort care	72 (23.0)
Insufficient communication about goals of treatment within the team	69 (22.0)
I was exposed to moral distress during the COVID-19 pandemic, *n* (%)	
Strongly agree	75 (24.0)
Somewhat agree	87 (27.8)
Do not know	23 (7.3)
Somewhat disagree	92 (29.4)
Strongly disagree	28 (8.9)
*Median (IQR)*	2 (2–4)
Level of moral distress was comparable to situation before COVID-19 pandemic, *n* (%)^a^	
Strongly agree	5 (3.1)
Somewhat agree	24 (14.8)
Do not know	11 (6.8)
Somewhat disagree	82 (50.6)
Strongly disagree	39 (24.1)
Major sources of moral distress*, *n* (%)^a^	
Work intensity—psychological exhaustion	27 (16.7)
Work intensity—physical exhaustion	23 (14.2)
Cooperation with not qualified colleagues	20 (12.3)
Changes in the standards of care	19 (11.7)
Severity of condition/prognosis of admitted patients	16 (9.9)
Personal interactions at the ICU	16 (9.9)
Prioritisation of care due to resource scarcity situation	16 (9.9)
Responsibility for insufficiently qualified colleagues	14 (8.6)
Work intensity—risk of infection	10 (6.2)
Organisational/institutional problems	6 (3.7)
Administration of experimental treatments	5 (3.1)

*COVID-19* Coronavirus disease, *IQR* interquartile range, *ICU* intensive care unit

^a^Percentage based on number of HCPs who answered ‘Strongly agree’ or ‘Somewhat agree’ on the question ‘I was exposed to moral distress during the Covid-19 pandemic’

*More than one answer possible

**Table 5:** Univariate analysis—potential predictors for moral distress

	*N*	Odds ratio (95% CI)	*p*-value
Sex	282		1.0000
Female versus Male		1.000 (0.606–1.650)	
Profession of HCPs	282		0.9835
Nurse versus Physician		1.005 (0.624–1.618)	
Age	282	0.998 (0.975–1.021)	0.8340
ICU experience	274	0.989 (0.965–1.014)	0.3863
Number of patients per HCP	270	1.072 (0.971–1.184)	0.1680
Type of hospital	268		0.2219
Secondary hospital		Ref	
Primary hospital		1.789 (0.848–3.773)	
University (tertiary) hospital		1.456 (0.853–2.485)	
Form of EOLD communication with patient’s family	280		0.3728
Family meeting		Ref	
Telephone call		1.377 (0.817–2.323)	
Videocall		0.953 (0.224–4.067)	
Meeting with patient’s family was not performed		2.860 (0.723–11.312)	
Involvement of the patient's family regarding EOLD	281		0.3810
Family/relatives were included in process, i.e. shared decisions		Ref	
Family/relatives were fully responsible for the decision		2.308 (0.421–12.648)	
Family/relatives were just informed about the decision; Patient was in comfort care despite family's disagreement		1.762 (0.747–4.159)	
Family/relatives were just informed about the decision; Patient was in comfort care if family/relatives did not disagree with the decision		1.175 (0.661–2.087)	
Family/relatives were not informed about decision		7.385 (0.880–61.994)	
Cannot answer as I was not involved in the decision process		1.108 (0.487–2.519)	
Inappropriate care of patient, Yes versus No	280	1.804 (1.082–3.008)	0.0237
Opinion about inappropriate care of patient respected, Yes versus No	165	0.511 (0.261–1.001)	0.0504
Therapy goals were always clearly explained and defined, Yes versus No	281	0.297 (0.160–0.551)	0.0001
Agreement with statement that the deaths of COVID-19 patients were dignified, Yes versus No	277	0.203 (0.114–0.359)	< .0001
Resource scarcity situation experience, Yes versus No	115	1.735 (0.807–3.732)	0.1585

*CI* confidence interval, *HCPs* health care providers, *ICU* intensive care unit, *COVID-19* coronavirus disease, *EOLD* end-of-life decision

**Table 6:** Multivariate analysis—predictors for moral distress

	*N*	Odds ratio (95% CI)	*p*-value
	276		
Inappropriate care of patient, Yes versus No		1.854 (1.057–3.252)	0.0312
Therapy goals were always clearly explained and defined, Yes versus No		0.515 (0.261–1.013)	0.0546
Agreement with statement that the deaths of COVID-19 patients were dignified, Yes versus No		0.235 (0.128–0.430)	< .0001

*CI* confidence interval; *COVID-19* coronavirus disease

## Discussion

This cross-sectional survey study describes palliative care practices and the burden of moral distress during the COVID-19 pandemic in the Czech Republic. This study is interesting through focusing research on both physicians and nurses. We found a significant shift in providing palliative care during the pandemic, in comparison with providing palliative care out of pandemic, that was also one of the sources of moral distress for HCPs. Our results also confirm that providing palliative care is more psychologically demanding from the point of view of HCPs than full treatment, which supports findings from other studies [27, 28].

The most important data from our survey show that more than half of all respondents were exposed to moral distress during the pandemic. However, only one-fifth of HCPs stated that the level of moral distress in the COVID-19 pandemic was comparable with the level of moral distress before the pandemic. These results are comparable with findings by similar studies focused on moral distress during the COVID-19 pandemic. More severe moral distress was described in HCPs working with COVID-19 patients compared to the level of moral distress in HCPs working with patients without COVID-19 in recent studies investigating mental health [1–3]. Contrary to these studies, our study was focused on the moral distress of HCPs working in ICUs, where critically ill patients were hospitalised. This care is extremely demanding for specific knowledge and hard and soft skills, especially in a new, more challenging situation such as the COVID-19 pandemic [29].

The following two factors were independently associated with moral distress risk: perception of inappropriate care and perception that the dying of COVID-19 patients was not dignified. These modifiable factors of moral distress are features of ethical climate and patient-oriented care.

Perceiving inappropriate care within the team regarding organ support was a major source of moral distress that more than half of HCPs experienced. This factor was reported in other studies, but our study confirms these findings in a larger study population [7, 8]. Inappropriate care is one of the most important triggers for moral distress. Improving the decision-making process at the EOL period and communication among all members of this process will reduce the incidence and the level of moral distress. This process requires further education and training for all HCPs. Correspondingly, after the DISPROPRICUS study, we know that improving the ethical climate is important for identifying patients receiving excessive care [30].

The authors consider it alarming that according to our respondents, one-fifth of patients did not die with dignity, even if dying with dignity should be the major goal of palliative care. The factors that led to this situation were mainly systemic problems, inconsistent physicians’ views on palliative care, and insufficient understanding of palliative care principles. Systemic problems will diminish after the pandemic has ceased, but the other factors need to be solved with proper education and protocols.

The interesting finding of the survey is the identification of the factor significantly associated with reducing the chance for the occurrence of moral distress. Dying with dignity reduced the chance for the occurrence of moral distress by approximately 76%. This is an important modifiable factor by HCPs involved in palliative care and ICU management by enhancing patient- and family-centred care. For example, ABCD (Attitude, Behaviour, Compassion, and Dialogue) approaches for dignity conserving care can be used in practice or for education purposes. Another essential part of palliative care is avoiding harm, respecting internal and external qualities, like autonomy of the patient, physical comfort, and reflecting the patient's spirituality. All conflicts among family relatives and HCPs must be prevented or solved effectively [10, 11]. The results show that the comfort of patients’ dying is very important, because it unquestionably affects the patients but also the HCPs’ moral distress in both manners.

Communication about potential inappropriate care and setting patients’ therapy goals is an essential part of palliative care. Our study found statistically significant differences between physicians and nurses in possible participation in these discussions. Moreover, our results indicate that despite both nurses and physicians expressing their opinion about inappropriate care, more than half of nurses’ opinions were ignored, which carries a high potential to trigger and worsen moral distress and burnout syndrome [27, 31]. Improvement in team communication, including treatment goals with candid and clear reasoning, could significantly reduce HCPs’ distress.

Poor communication could also be caused by missing local protocols for providing palliative care and limited availability of palliative teams in hospitals. As mentioned in the results, only less than half of the respondents had available dedicated palliative team in their hospital, and only one-third had local protocols for providing palliative care**.** This is the hospital's responsibility and ICU management, as other studies investigating the role of palliative teams in ICU palliative care showed [32, 33]. Establishing a palliative care team in each hospital is a long-term goal. Additionally, improving palliative care education, creating and implementation of local protocols for palliative care practice are one of the major goals of the newly established palliative initiative in the Czech Republic (https://www.med.muni.cz/ripe-icu/en).

The EOLD is made within the shared decisions model based on the patient's actual health state and prognosis, but also the patient's wishes and values. One way to acknowledge them is through discussion with the patient. In the ICU environment, this is often impossible. The legal way to know the will of patients unable to communicate sufficiently is through advanced directives. Our findings suggest that this possibility is not used adequately in the Czech Republic, probably because it was legalised in 2012 and has not been well known throughout the population yet.

EOLD often involved physicians, primary nurses, and patients’ relatives, but in general, nurses were surprisingly involved in only half of the reported cases. These findings are opposite to the whole teamwork concept in the ICU and contrary to the overall recommendations regarding EOLD [34, 35]. The authors emphasise that the opinion of nursing staff should be taken into account. Similar results were confirmed in more studies [8, 9]. These facts represent an opportunity to improve the ICU atmosphere and reduce the risk of moral distress, especially among nurses.

Another important finding from our study is that only approximately one quarter of respondents experienced family involvement as an essential part of the shared decisions model. We have no data to compare practices regarding the involvement of relatives in the EOL process before the pandemic. On the other hand, COVID-19 and related restrictive public and visitation policies separated patients from their beloved ones, leaving them alone. These factors are reported to be associated with disturbed experience and increased risk of posttraumatic stress disorder in family members [36, 37]. But surprisingly, our study findings show that the vast majority of relatives (85.9%) in the cases where HCPs communicated about EOLD with family were informed personally. It was reported that visitation was totally banned during the pandemic or allowed only for EOL visits [37]. Early family involvement and the possibility of psychological support during EOLD for relatives and HCPs are strongly recommended, but during extraordinary times of COVID-19, family members were loaded by additional frustration [27, 38, 39].

One of the key principles of palliative care is to relieve patients’ discomfort; therefore, monitoring symptoms is essential for efficient care. Our findings show that the overall monitoring of specific forms of distress could be improved. Spiritual distress was the least monitored, and our results correspond to other studies investigating spiritual distress, its monitoring, and possible problems. But this kind of distress is the most complicated to evaluate. The HCPs are often unsure how to approach these topics or maybe scared of possible patients’ questions [40, 41]. The professionalism regarding this sometimes overlooked topic requires proper education, clinical practice, and extensive training; therefore, it is impossible to have qualified HCPs at each ICU. For this reason, at least one member of the palliative team should be qualified in spiritual distress monitoring and available to assist ICU staff.

According to our results, the practice of palliative care during the pandemic was different. The reported differences were mainly regarding insufficient communication among all HCPs and relatives, providing palliative care by less skilled HCPs, lack of time for patients and for psychological or spiritual support. The most frequent causes of this difference were health system congestion, personnel factors [9], clinical characteristics, and the prognosis of COVID-19 infection [4, 5, 7].

The COVID-19 pandemic exposed all HCPs to the new situations. Physicians had to perform rationing and triage patients without enough information about this new diagnosis with a poor prognosis [42–44]. The hospitals were overloaded, and the nursing staff had less time for patient care, which could lead to poor quality of care. Lack of equipment, medicaments, or less time represented the situation called resource scarcity. COVID-19 pandemic showed the problem with resource allocation and the potential risk of inappropriate care. Discussion about resource allocation and developing rules and guidelines is essential, and it can prevent moral distress development in medical staff in the future [12, 13].

As we know, the level of moral distress represents an essential factor for the risk of burnout or job dissatisfaction and potential turnover and attrition in the profession. Many sources and modifiable factors of moral distress have been mentioned. It is a challenge and commitment for the management of hospitals and ICUs at the local level and, of course, a challenge at the national level for the direction of the Ministry of Health and all stakeholders involved (e.g. universities, professional societies).

### Strength and limitations of the study

The study's main strength is a nationwide cross-sectional study that includes both physicians and nurses. The second main strength is that the study was supported by the new officially established palliative initiative in the Czech Republic and supported by the Ministry of Health and professional societies of HCPs. This study has several limitations. First, the survey was performed only in the Czech Republic; hence, it may not be generalised to other countries. The second limitation is the low response rate. Only 14.5% of respondents opening the survey finished it completely. Another possible limitation is the respondents’ composition. Some of them worked in specially established ICUs without previous experience with palliative care; on the other hand, the survey describes the real providing palliative care during the pandemic. The national guidelines and recommendations for palliative care practice are another possible limitation. These guidelines were published in 2010 without any recent revision, despite palliative care development. Still, both groups (from newly established ICUs and ‘traditional’ ICUs) reported problems with moral distress. We therefore assume that our work is meaningful and will need to be continued in the long term.

## Conclusion

According to our survey findings, palliative care was changed during the COVID-19 pandemic in the Czech Republic and represented a challenge for all HCPs with a possible risk of moral distress development. The major sources were perception of inappropriate care and perception patients' dying with inadequate dignity. Improvement of the decision-making process and communication at the end of life could lead to a better ethical and safety climate.

## Supplementary Information

**Additional file 1.** **Appendix 1.** Questionnaire.

**Additional file 2** **Appendix 2.** Complete analysis.

**Additional file 3** **Appendix 3.** Source data.

## Data Availability

All data used and analysed during this study are available in Attachments.

## Abbreviations

COVID-19: Coronavirus disease 2019
EOL: End of life
EOLDs: End-of-life decisions
HCPs: Health care providers
ICU: Intensive care unit
IQR: Interquartile range
RIPE-ICU: Research Initiative in Palliative Care and Ethics in Intensive Care Unit
